# KASABACH MERRITT SYNDROME: MANAGEMENT WITH INTERFERON

**DOI:** 10.4103/0019-5154.70705

**Published:** 2010

**Authors:** Sandhya Acharya, Kalyani Pillai, Abel Francis, S Criton, V K Parvathi

**Affiliations:** *From the Department of Dermatology, Amala Institute of Medical Sciences,(AIMS), Thrissur, Kerala, India*

**Keywords:** *Kasabach Merritt syndrome*, *interferon α 2b*, *coagulopathy*

## Abstract

Kasabach Merritt Syndrome (KMS) is a rare, locally aggressive, vascular tumor. The objectives of treatment of KMS are to prevent bleeding from consumptive coagulopathy and induce vascular tumor regression. A 14-month old female child was brought with a reddish lesion on the left scapular area noticed at birth, which suddenly increased in size since 3 days. Hemogram revealed anemia severe thrombocytopenia, prolongation of bleeding, clotting time and increased fibrin degradable products, suggestive of KMS. Coagulopathy was managed by transfusing fresh frozen plasma and platelets. Oral prednisolone up to 5mg/kg/day for four weeks yielded no effect on thrombocytopenia or regression of tumor size. Embolization of feeding artery was attempted but not feasible. We used Interferon –alpha– 2b (IFN α 2b), in a dosage of 3million IU/m^2^ /day subcutaneously. Within a month the platelet count increased and the vascular tumor started regressing. This case signifies the importance of step wise management of KMS.

## Introduction

Kasabach Merritt Syndrome (KMS) is a rare, locally aggressive, vascular tumor. It is characterized by a rapidly enlarging vascular anomaly, consumptive coagulopathy, thrombocytopenia, prolonged PT and APTT, hypofibrinogenemia, the presence of D dimer and fibrin split products with or without microangiopathic hemolytic anemia.[1] Kasabach Merritt Syndrome was first noted by Kasabach and Merritt in 1940.[2] It’s clinical characteristics and histopathology are distinct from those of hemangiomas. Histopathology usually reveals a tufted angioma or Kaposiform hemangioendothelioma.[1,3,4] Prognosis is poor due to few treatment options.

A variety of treatments have been used to induce tumor regression, including prednisolone, dipyridamole, pentoxyfylline, ticlopidine, and heparin, interferon (IFN), vincristine, radiotherapy, and embolization of the feeding vessel to the tumor. We followed a step-wise regimen as evaluated by S. Wananukul *et al*.[5]

## Case Report

**Figure 1:**
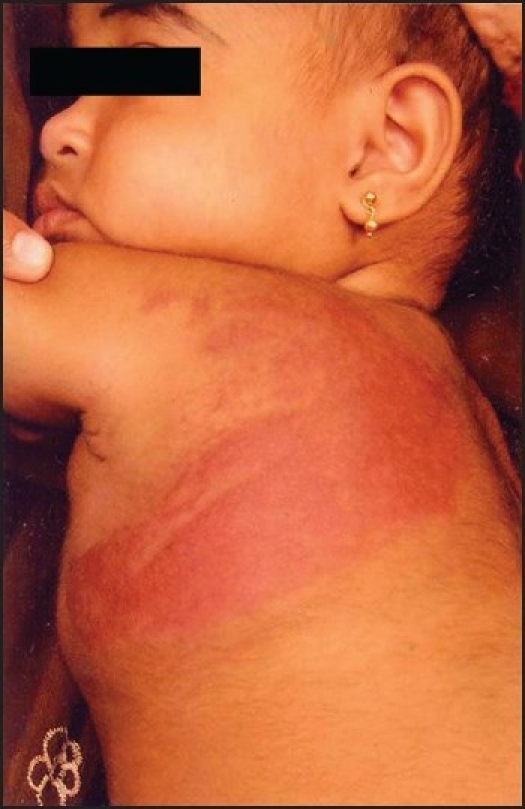
Hemangioma at eight months of age

**Figure 2:**
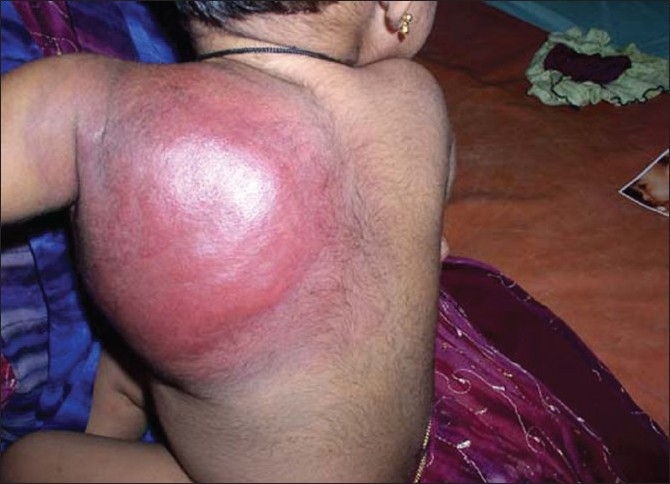
Hemangioma at one year two months of age

A 14-month-old female child came with history of sudden increase in size of lesion over left shoulder, back and chest wall associated with severe pain. This lesion was present from birth [Figure 1]. On clinical examination the baby had petechiae over the face, a large hemangioma over left scapular area extending on to the left axilla and left mammary area [Figure 2]. The lesion was warm, tender with peau de orange appearance. There was no bruit over the lesion. Her systemic examination was normal. Our presumptive diagnosis was KMS and we investigated accordingly [Table 1].

**Table 1:** Investigations

Platelet count	25,000cells/cumm
BT	>30 min
CT	15 min
PT	15/12 min
APPT	52/32 min
FDP (D-dimer)	2610 (n<500 ng/ml)

A hemogram revealed anemia with severe thrombocytopenia and prolongation of bleeding and clotting time and the presence of increased fibrin degradation products. Skin biopsy of the lesion showed dilated irregular thin walled vascular channels lined by flattened endothelial cells in the dermis. Ultrasonography of the chest revealed large ill defined heterogeneous solid diffuse lesion noted over the left side of the upper chest wall extending from front to back and axilla, involving skin and subcutis. Color Doppler showed feeding arterial supply from the intercostals vessels.

**Figure 3:**
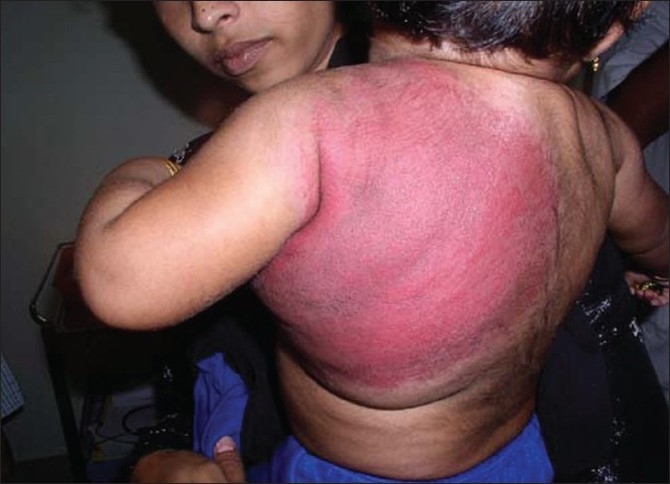
Two months after steroids and interferon

**Figure 4:**
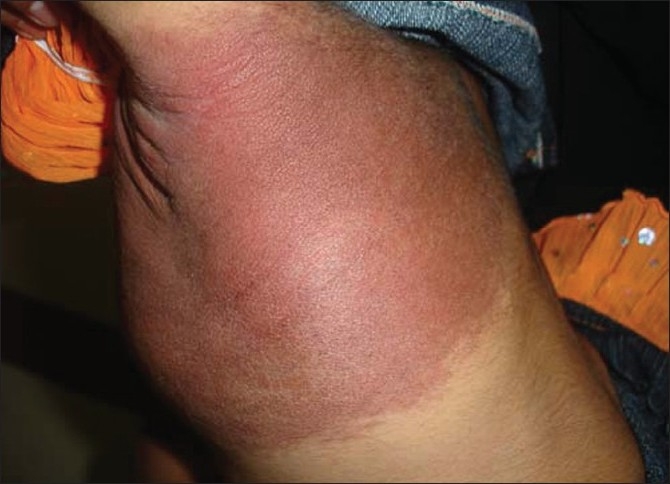
One year after interferon therapy

With a confirmed diagnosis of KMS, the patient was started on transfusions of platelets and fresh frozen plasma as she had oral bleeds. Oral prednisolone was started 2mg/kg body weight to suppress the hemangioma. Although the coagulopathy reverted to normal in a few days, platelets started decreasing and so she was referred to higher centre for thrombosing the feeding vessels of the hemangioma. However, she was referred back to us as it was not feasible. In spite of increasing the dose of steroids to 5mg/kg body weight, the platelet count continued to drop. It was then that we started her on interferon α2b in a dose of 3million IU/m^2^ /day subcutaneously. Within four weeks her platelet counts became normal and the hemangioma started regressing [Figures 3 and 4].

## Discussion

KMS is a rare syndrome. The objectives of treatment of KMS are to prevent bleeding from thrombocytopenia and consumptive coagulopathy and to induce vascular tumor regression. Thrombocytopenia in KMS is due to peripheral destruction, as platelets are trapped in these vascular tumors. Platelet transfusions can also exacerbate bleeding.[7] Platelet transfusions should therefore be used only when bleeding is clinically apparent as in our patient.

Tumor regression can be induced by various treatment modalities, including prednisolone,[1,3,5] dipyridamole, pentoxyfylline,[1] ticlopidine,[1] and heparin.[1] IFN,[9] vincristine, radiotherapy, and embolization of the feeding vessel to the tumor.[6–8] Steroid was used as first line therapy because it is available at a relatively low cost. IFN α 2b was used as second line therapy because of its known efficacy, although the cost of the treatment was considerably higher than that of the first line drugs.

Our patient had complications on high dose prednisolone, particularly hypertension and cushingoid appearance. Nevertheless, because of its low cost and ease of administration, steroid may still be most appropriate first line drug for the initial few weeks of therapy.

IFN has been used in the treatment of KMS since 1992 due to its antiproliferative and antiangiogenic effect.[9] In some series, IFN has been recommended as first line therapy The two forms of IFN α 2a and IFN α 2b, are probably identical in efficacy, and both forms have been used in KMS and life- threatening hemangiomas of infancy at a dosage of 3 million IU/m^2^/day, with favorable results.

The known adverse effects of IFN therapy include fever, neutropenia, mild anemia, mild elevation of liver function test, and, most deleteriously, spastic diplegia in infancy.[10] In conclusion, KMS may be treated in a stepwise approach. High dose steroid is probably the most cost effective, but does not result in a high rate of response and is not tolerated well. Response to IFN is more favorable, but life threatening adverse events can occur.
